# A review of digital orthopedic techniques in pre- and intra-operative management of scaphoid fracture

**DOI:** 10.1530/EOR-2026-0084

**Published:** 2026-10-01

**Authors:** Shuai Wang, Zhe Yi, Wei Chen, Bo Liu

**Affiliations:** ^1^ Department of Hand Surgery, Beijing Jishuitan Hospital, Capital Medical University, Beijing, China; ^2^ Department of Hand Surgery, Beijing Jishuitan Hospital, Peking University Fourth School of Clinical Medicine, Beijing, China; ^3^ Beijing Research Institute of Traumatology and Orthopaedics, Beijing, China

**Keywords:** scaphoid fracture, digital orthopedics, artificial intelligence, finite element analysis, three-dimensional printing, computer-assisted navigation, robot-assisted surgery

## Abstract

- Digital orthopedics integrates artificial intelligence, finite element analysis, virtual surgical planning, intraoperative navigation, robotics, and three-dimensional printing into a precise, individualized workflow for scaphoid fracture management.
- Artificial intelligence-based algorithms achieve high diagnostic accuracy for scaphoid fractures on plain radiographs, aid in detecting occult fractures, and support treatment decisions.
- Three-dimensional fracture mapping and finite element analysis enable quantitative assessment of fracture morphology and biomechanical stability, supporting individualized preoperative planning.
- Computer-assisted navigation reduces radiation exposure and guidewire attempts compared with conventional fluoroscopy but remains limited by registration inaccuracy and dependence on manual execution.
- Robot-assisted surgery improves procedural consistency and screw accuracy, with a learning curve of approximately 10–20 cases; however, it is constrained by longer setup times, high costs, and predominantly level IV evidence.
- Virtual surgical planning combined with three-dimensional printing provides patient-specific percutaneous guides, reduction templates, anatomical models, and custom prostheses, improving accuracy, reducing radiation exposure, and enabling motion-preserving salvage for scaphoid nonunions and scaphoid nonunion advanced collapse.
- Current evidence is largely based on small case series and cadaveric studies, highlighting the need for standardized protocols and high-quality comparative trials to support widespread adoption.

## Introduction

Scaphoid fractures represent a significant clinical challenge, accounting for 60–90% of all carpal fractures and 2–7% of all fractures (1, 2). Despite the high frequency of scaphoid fractures, achieving timely and accurate diagnosis followed by appropriate management remains difficult. Although many scaphoid fractures heal uneventfully with appropriate treatment, delayed diagnosis, inadequate treatment, or established nonunion can lead to clinically important complications, including scaphoid nonunion and subsequent scaphoid nonunion advanced collapse (SNAC) wrist (3). Even with surgical intervention, patients may suffer from debilitating pain and functional impairment, highlighting the critical need for precise and effective treatment strategies.

The evolution of orthopedic surgery has been marked by several transformative technological waves, including microsurgery and minimally invasive surgery (MIS). The latest revolution is driven by digital orthopedics, an emerging interdisciplinary field that integrates computer science with orthopedic practice (4). It encompasses all digital medical technologies applied to the research, diagnosis, treatment, and rehabilitation of musculoskeletal disorders. In selected settings, digital techniques may support more precise imaging assessment, patient-specific planning, and more reproducible intraoperative execution; however, many applications remain investigational or in early clinical adoption rather than routine mainstream practice.

This narrative review aims to synthesize the current evidence and elucidate the role of key digital orthopedics technologies, specifically artificial intelligence (AI)-assisted scaphoid fracture diagnosis, finite element analysis (FEA), three-dimensional (3D) reconstruction and fracture mapping, computer-assisted navigation, robot-assisted surgery, and 3D printing, in the pre- and intra-operative management of scaphoid fractures. By examining their technical principles, clinical applications, reported outcomes, and inherent limitations, this review seeks to provide a comprehensive overview of the contemporary digital surgical landscape for scaphoid fractures and to identify future directions for research and clinical integration.

## Methods

This narrative review was conducted to synthesize and critically appraise the contemporary literature on the application of digital orthopedic techniques in the pre- and intra-operative management of scaphoid fractures.

A comprehensive literature search was performed across three major electronic databases: PubMed, Web of Science, and Scopus. The search strategy used a combination of keywords and Medical Subject Headings (MeSH) terms related to digital technologies and the scaphoid. The core search string was: (‘3D’ OR ‘three-dimensional’ OR ‘3-dimensional’ OR ‘computer’ OR ‘robot’ OR ‘artificial intelligence’ OR ‘AI’ OR ‘deep learning’ OR ‘machine learning’ OR ‘convolutional neural network’ OR ‘CNN’ OR ‘virtual simulation’ OR ‘finite element analysis’ OR ‘planning’ OR ‘guide’ OR ‘guidance’ OR ‘navigate’ OR ‘navigation’) AND (‘scaphoid’). No restrictions were placed on publication year to capture the full evolution of these technologies; the search was limited to articles published in English.

Included studies comprised clinical series (retrospective or prospective), randomized controlled trials, cadaveric studies, and technical notes that provided substantive methodological or outcome data. Case reports were included for novel or first-in-human applications to illustrate the scope of technological innovation. Studies addressing other carpal bones without specific scaphoid data were excluded.

Data from included studies were extracted into standardized tables to facilitate comparison and synthesis. Extracted items included study design, sample characteristics (patients or cadavers), digital technology, surgical procedure, comparator (if any), and primary outcomes for accuracy (e.g. screw deviation, reduction accuracy), efficiency (e.g. operative time, fluoroscopy usage), safety, and clinical results (e.g. union rate, functional scores). Findings were then organized by technology type, and implications, advantages, and limitations were summarized narratively to provide an integrated overview of the current state and future trajectory of digital orthopedics in scaphoid surgery. This is further illustrated in Fig. 1 as a chronological timeline of key developments.

**Figure 1:**
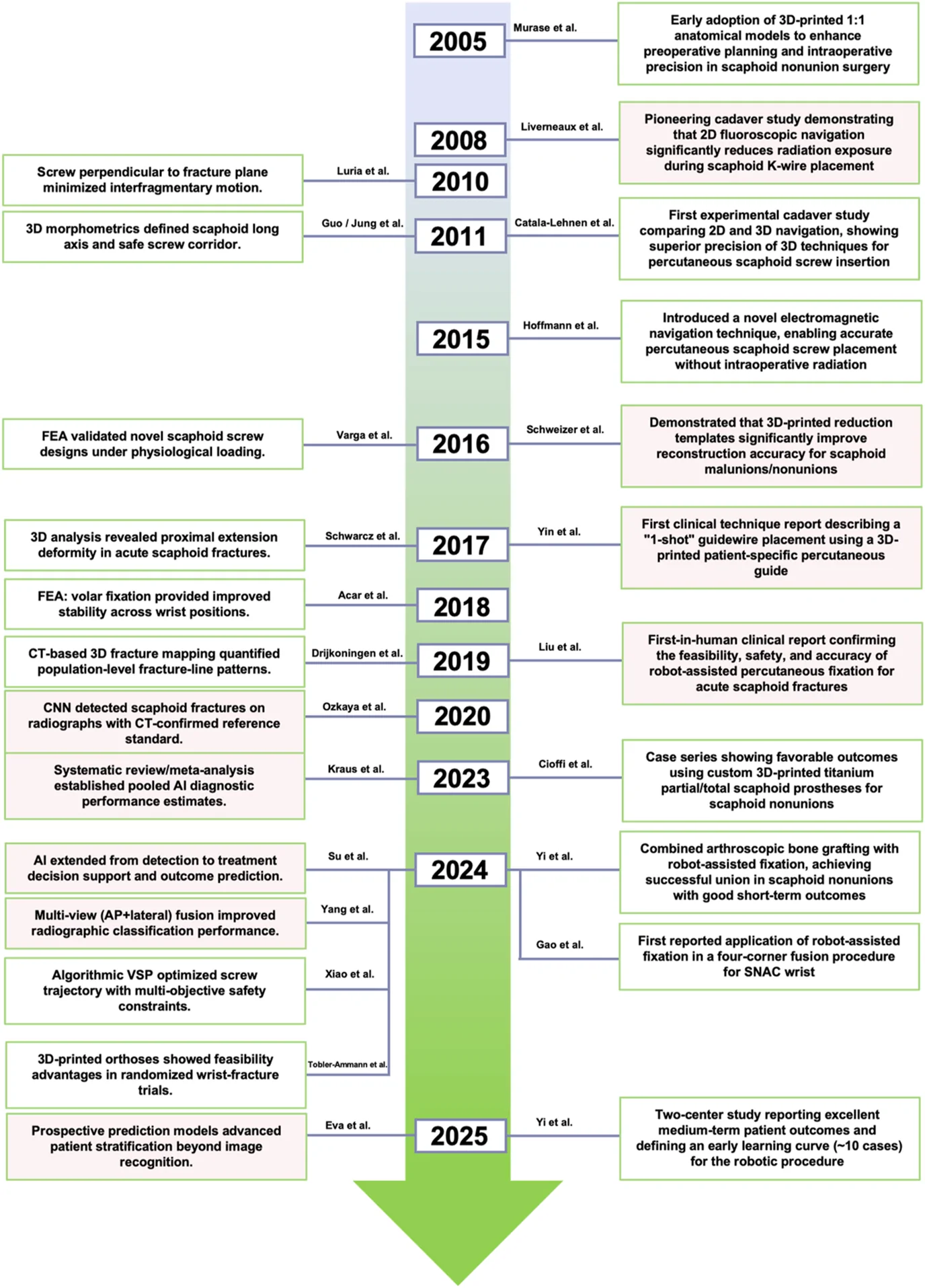
Key events in the exploration of digital orthopedics techniques in scaphoid fractures were retrospectively shown from 2003 to the present. The left and right sides of the arrow represent pre- and intra-operative applications, respectively. The text box with a pink background shows the crucial research. SNAC, scaphoid nonunion and advanced collapse.

## Preoperative management

### AI-enabled scaphoid fracture detection

AI, particularly deep learning-based algorithms, is increasingly being investigated as an adjunct in medical imaging analysis for scaphoid fracture diagnosis. It may improve diagnostic efficiency and accuracy in selected settings while also supporting progression from assisted recognition toward clinical decision support; however, routine clinical adoption remains limited and requires robust external validation.

#### Convolutional neural networks (CNNs)

CNNs are a core deep learning method for image recognition, with architectures inspired by the functional organization of the biological visual cortex. The overall CNN workflow used in this review is summarized in Fig. 2 to provide a schematic overview of the data input, feature extraction, and prediction pipeline. Using stacked convolutional and pooling layers, CNNs automatically learn features from large-scale image data, ranging from low-level patterns (e.g. edges and textures) to high-level representations (e.g. anatomical structures and fracture lines). For scaphoid fracture diagnosis, CNNs are typically trained on large wrist radiograph datasets annotated by experienced radiologists or orthopedic surgeons, including fracture and non-fracture cases. During training, models learn subtle manifestations such as minor cortical disruption, indistinct fracture lines, trabecular architecture changes, and mild density alterations, enabling scaphoid fracture identification that can, in some settings, approach or exceed expert performance. Studies support the feasibility and effectiveness of CNN-based radiographic diagnosis: Yang *et al.* (5, 6) validated automated detection and classification; Yoon *et al.* (7) developed and validated a deep learning model for radiographic identification; Hendrix *et al.* reported (8, 9) multi-view performance comparable to that of experienced musculoskeletal radiologists; and Yang *et al.* (5) improved detection and classification by integrating anteroposterior (AP) and lateral (LA) views, highlighting AI potential in more complex scenarios.

**Figure 2:**
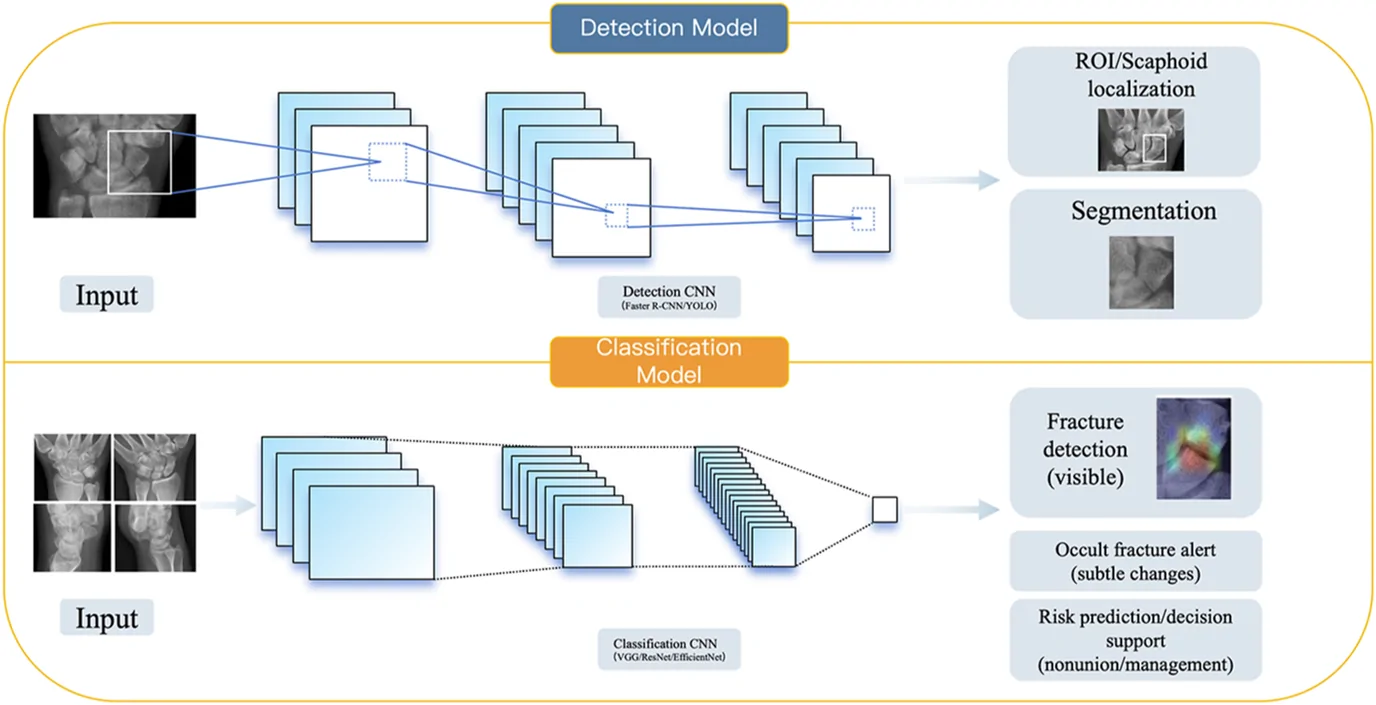
Schematic overview of a convolutional neural network-based system for automatic detection.

#### Diagnostic performance of AI models

To more objectively evaluate the clinical value of AI for scaphoid fracture diagnosis, multiple systematic reviews and meta-analyses have synthesized and appraised the evidence (5, 6, 7, 8, 9, 10, 11, 12, 13, 14, 15, 16, 17, 18, 19, 20, 21, 22, 23, 24, 25, 26). Orji *et al.* (15) reviewed AI and machine learning applications in this field, whereas Kraus *et al.* (21) and Oeding JF *et al.* (16) provided higher-quality evidence using more rigorous systematic review and meta-analytic methods. Overall, these reviews reached broadly consistent conclusions: commonly used AI models achieve high diagnostic accuracy, sensitivity, and specificity for scaphoid fractures, with many metrics exceeding 90%. The study by Ozkaya *et al.* (14) likewise showed that the AI system performed stably on real-world clinical data. For clarity, Table 1 summarizes key information from selected representative studies. However, CNN performance should be interpreted cautiously. Important limitations include dependence on training-data quality and case mix, spectrum and selection bias, limited external validation, imperfect explainability, and sensitivity to imaging-acquisition protocols. Reported model-level accuracy should therefore not be equated with clinical readiness, and CNN output should support rather than replace expert assessment (22).

**Table 1:** Artificial intelligence for scaphoid fracture-related tasks on radiographs: study characteristics and performance.

Study	Study design	AI model	Dataset size	Performance metrics	Reference standard (ground truth)
Eva *et al.* (20)	Retrospective diagnostic study	Transfer-learning CNN (VGG)	300 radiographs (train 200; test 100)	Test: AUC 0.77; Sens 0.84; Spec 0.60; Acc 0.72	CT reference standard (visible vs occult)
Ozkaya *et al.* (14)	Retrospective; AP radiographs	CNN classifier (backbone NR)	390 patients	Sens 0.76; Spec 0.92; AUC 0.84; F1 0.83	CT-confirmed diagnosis
Yoon *et al.* (10)	Retrospective development + validation	Two-stage: Cascade R-CNN (ROI) + EfficientNetB3	11,838 images (train 8,356; val 1,177; test 2,305)	Apparent: AUC ∼0.96; Sens 0.87; Spec 0.92. Occult: AUC 0.81; 20/22 detected	Radiology interpretation + confirmatory imaging (repeat XR/CT/MRI) + follow-up
Hendrix *et al.* (9)	Retrospective development/validation + observer study	Two-stage: scaphoid segmentation CNN + detection CNN	Train: 1,039 (seg) + 3,000 (det); Test: 190 images	Test: AUC 0.87; Sens 0.78; Spec 0.84	Follow-up CT within 4 weeks for test set (ground truth)
Hendrix *et al.* (8)	Retrospective multi-view; reader study; external cohort	Detection model on multi-view hand/wrist radiographs (backbone NR)	Detection: 4,316 + 688 images (two centers); plus 12,990 + 1,117 for view/side tasks	AI: AUC 0.88; Sens 0.72; Spec 0.93	PACS/EHR final diagnosis (not restricted to CT/MRI)
Li *et al.* (18)	Retrospective	YOLOv3 (ROI) + MobileNetV3 (classifier)	1,918 radiographs (600 patients)	Sens 0.82; Spec 0.94; AUC 0.92	Reference labels by 4 hand surgeons (majority vote)
Yang *et al.* (6)	Retrospective method study; 5-fold CV	Faster R-CNN (ROI) + FPN/attention classifier	361 images (167 fx; 194 normal)	AUC 0.92; Sens 0.79; Spec 0.90; Acc 0.85	Surgery-verified fractures
Kraus *et al.* (21)	Systematic review + DTA meta-analysis	Multiple CNNs (review)	10 studies; 7 in meta (3,373 images)	Pooled: Sens 0.80; Spec 0.89; AUC 0.88	As reported across included studies (often CT/MRI)
Lee *et al.* (19)	Clinical validation	AI model for scaphoid + other fractures (backbone NR)	Train 4,432 images; test 593 subjects	Scaphoid: AUC 0.81	Two experts labeled fractures (bounding boxes)
Oeding *et al.* (16)	Systematic review	Multiple CNNs (review)	21 studies; 8 scaphoid-focused	Scaphoid AUROC range 0.72–0.93 (across studies)	As reported across included studies
Yang *et al.* (5)	Retrospective method study; multi-view fusion	Two-stage ROI + multi-view CNN fusion	175 cases (AP + lateral pairs)	Acc 0.90; Recall 0.87; Precision 0.90 (classification)	Surgery-confirmed fractures; annotated by 2 physicians
Eva *et al.* (26)	Prospective clinical prediction model	ML risk model (5 algorithms; non-CNN)	422 patients; 117 true fx (18 occult)	AUC 0.77; Brier 0.159; calibration slope 0.84	Fracture confirmed on CT/MRI or follow-up radiographs
Tümen *et al.* (12)	Retrospective prognostic study	Deep learning on pre-op radiographs (TensorFlow)	346 nonunions (263 success; 83 failure)	DL Acc 0.94 (reported); logistic regression Acc 0.66	Union outcome ≥3 months (healed vs not healed)

Acc, accuracy; AUC/AUROC, area under the receiver operating characteristic curve; CV, cross-validation; ED, emergency department; F1, F1-score; fx, fracture; MSK, musculoskeletal; NR, not reported; ROI, region of interest; Sens, sensitivity; Spec, specificity; Val, validation. Reference standard (ground truth) indicates how labels/outcomes were established (e.g. CT/MRI, surgery, follow-up, chart review, expert consensus).

#### AI-assisted occult fracture detection

Occult scaphoid fractures are highly prone to missed diagnosis and delayed treatment. In the acute phase, fracture lines are often subtle, typically without obvious displacement, and conventional plain radiography may have only 70–80% sensitivity. Using deep learning, AI can detect subtle fracture-related imaging changes that are difficult for the human eye to perceive, improving detection. A clinical validation study by Yoon *et al.* (7) showed that AI assistance significantly increased orthopedic surgeons' diagnostic sensitivity for occult scaphoid fractures, supporting its clinical value. Comparing model performance with experts, Bützow *et al.* (25) found that the model outperformed experts in an occult-fracture cohort, detecting 41% of occult scaphoid fractures. Langerhuizen *et al.* (20) assessed whether deep learning could reach human observer performance for detecting radiographically visible and occult scaphoid fractures; their results supported AI adoption and suggested that it may be a key tool for this diagnostic challenge. This likely reflects AI’s fine-grained, stable analysis of pixel-level information, enabling the capture of subtle patterns that may be missed because of limited experience or visual fatigue.

At present, AI applications in scaphoid fracture care are shifting from binary fracture classification toward clinical decision support systems (CDSS) embedded in complex workflows. First, AI is extending into treatment-related steps for scaphoid nonunion. Tmen *et al.* (12) explored deep learning for scaphoid nonunion management, including radiomics-based nonunion risk prediction, fragment vascularity assessment, and support for planning bone grafting and internal fixation. Second, AI is increasingly used to generate treatment recommendations. Su *et al.* (13) reported a model that not only identified scaphoid fractures with high accuracy but also automatically recommended operative versus nonoperative management or predicted nonoperative outcomes from imaging features (e.g. fracture type, displacement, and comminution), indicating a shift from diagnosis to decision support. However, several issues remain before widespread implementation. Wilson (11) emphasized that AI must demonstrate tangible clinical utility (e.g. reducing clinicians' cognitive burden and improving outcomes), narrow the trust gap, establish ethical and regulatory frameworks, and clarify responsibility boundaries. A critical review by Miller *et al.* (17) further underscored the need to define legal liability in AI-assisted decision-making, implement continuous algorithm quality control and oversight, and mitigate algorithmic bias before large-scale deployment.

### 3D visualization and biomechanical simulation

Following AI-based initial imaging diagnosis, digital orthopedics enables deeper analysis. Using 3D visualization and biomechanical simulation, clinicians can move beyond two-dimensional (2D) imaging to perform multi-angle, quantitative assessment of scaphoid fractures, shifting from seeing the fracture line to understanding fracture characteristics and providing stronger anatomical and biomechanical evidence for individualized treatment planning.

#### Precision fracture phenotyping

Thin-slice CT-based segmentation and 3D reconstruction (typically ≤1 mm slice thickness) reduce osseous overlap and depict scaphoid anatomy and fracture morphology at an approximately 1:1 scale, enabling more reliable identification of fracture-line trajectories, fragment relationships, and features such as comminution and impaction and providing a foundation for quantitative analysis and surgical planning. At the population level, 3D fracture mapping and density mapping register and overlay multiple 3D fracture models onto a template scaphoid and visualize high-frequency regions of fracture lines or surfaces using color-density encoding. Using density mapping, Drijkoningen *et al.* (27) identified a common high-incidence region for acute scaphoid fractures in the middle third and summarized fracture-trajectory orientations for proximal–middle versus distal fractures. In a multicenter CT cohort, Turow *et al.* (28) mapped fracture lines and comminution zones onto a unified scaphoid model and showed that comminution preferentially clusters in specific regions, including the dorsal ridge and volar scaphoid waist, facilitating injury-pattern interpretation and informing imaging-assessment priorities. At the individual level, 3D models with standardized coordinate measurements can quantify displacement and rotation that are difficult to assess consistently on 2D imaging. Using a 3D coordinate system to compare displaced versus nondisplaced acute scaphoid waist fractures, Schwarcz *et al.* (29) found that typical displacement is dominated by proximal fragment extension and proposed that acute humpback deformity is better explained as proximal extension deformity, supporting reduction strategies that prioritize correcting the proximal fragment.

#### Finite element analysis (FEA)

3D visualization primarily reconstructs anatomical structures for intuitive characterization of fracture morphology, whereas FEA evaluates fracture stability mechanically and compares fixation strategies. FEA discretizes scaphoid geometry into small elements, assigns material properties (e.g. elastic modulus and Poisson’s ratio), and simulates stress and deformation under physiological loads (e.g. grip forces and weight bearing) in a virtual environment, integrating morphology and mechanics into a quantitative assessment framework. Methods and key findings from recent scaphoid FEA studies are summarized in Table 2 to facilitate comparison. FEA can elucidate fracture mechanisms and quantify stability. Santoshi *et al.* (30) simulated loading across different wrist positions and reproduced scaphoid waist fracture development, suggesting relative mechanical vulnerability of this region. In individual cases, FEA can quantify interfragmentary micromotion; excessive micromotion implies higher nonunion risk, enabling numerical assessment of fracture stability. FEA also can perform virtual stress testing of internal fixation constructs to support selection of an appropriate stabilization strategy. Using the same fracture model, Acar *et al.* (31) compared stability and screw stress distributions for volar versus dorsal screw insertion. Acar *et al.* (32) further compared single-screw with dual-screw fixation, suggesting that comminuted or high-risk fractures may require more robust constructs. In a transverse scaphoid waist fracture model, Srivastav *et al.* (33) compared single-screw fixation, screw plus Kirschner wire (K-wire), and dual K-wire fixation, reporting that FEA can inform construct selection and optimize screw or K-wire orientation and relative positioning, providing quantitative guidance for early postoperative rehabilitation and intraoperative technical refinement. FEA can also validate novel internal fixation designs: Varga *et al.* (34) used FEA to assess a new scaphoid screw, reducing trial and error and accelerating clinical translation, and Luria *et al.* (35) suggested that FEA can help identify more suitable fixation options for acute fractures, improving therapeutic decision-making accuracy.

**Table 2:** Summary of finite element studies on scaphoid waist fracture.

Study	Study aim	FE model & fracture type	Fixation / variables	Key FE outputs (units)	Main conclusion
Luria *et al.* (35)	Compare fracture-perpendicular vs scaphoid long-axis screw strategies	CT-based 3D scaphoid; Herbert B1/B2/B3 patterns	Headless screw trajectory: perpendicular vs central long axis	Interfragmentary motion (mm); screw/thread strain (microstrain, με)	Perpendicular trajectory minimizes fracture motion
Varga *et al.* (34)	Evaluate two novel screw designs for rotational stability under functional loading	FE waist fracture/osteotomy scaphoid model	Conventional vs two novel screws; wrist positions; compression force (N)	Interfragmentary motion (mm), incl rotational component; acting moments (N·mm)	Novel designs reduce rotational IFM mainly when compression is low
Acar *et al.* (31)	Compare volar vs dorsal screw fixation biomechanics	FE Herbert B2 transverse waist fracture	Headless screw via volar vs dorsal approach; TE/N/TF wrist positions	Gap displacement (mm); rotation (deg); von Mises stress (MPa)	Volar fixation yields lower displacement/rotation across positions
Acar *et al.* (32)	Compare single vs double screw constructs (FE + preliminary clinical series)	FE Herbert B2 waist fracture; clinical nonunion cohort reported	Single 3.5-mm screw vs two parallel 2.5-mm screws; wrist positions	Gap displacement (mm); rotation (deg)	Double screws improve construct stability vs a single screw
Santoshi *et al.* (30)	Investigate mechanism of scaphoid waist fracture as a function of distal load site	CT-based FE (12 wrists); waist fracture formation prediction	Distal loading site along STT region; defined proximal supports	Predicted failure site; principal stress (MPa); tension vs compression cortex mode	Load location governs cortical failure mode (tension vs compression)
Srivastav *et al.* (33)	Compare immediate stability of common postoperative implant configurations	CAD/FE transverse waist fracture; compression and distraction loading	Single screw (directions), screw + K-wire (layouts), two K-wires (layouts)	Total deformation (mm); factor of safety (unitless)	Best stability: posteriorly directed screw; parallel screw + K-wire; divergent K-wires

IFM, interfragmentary motion; TE/N/TF, total extension/neutral/total flexion; STT, scaphotrapeziotrapezoid. Units are standardized as shown: displacement/IFM in mm, compression force in N, moments in N·mm, stress in MPa, rotation in degrees (deg), strain in microstrain (με), and factor of safety is unitless. Where a study reported an output qualitatively, the wording reflects that reporting.

The integration of 3D visualization with FEA enables a preoperative workflow bridging morphology and mechanics. Fracture mapping and patient-specific 3D analysis characterize population patterns and individual features, whereas FEA builds on these data to interpret the mechanical implications of morphology. 3D visualization quantifies comminution and displacement more accurately, and because these features influence stability, FEA can further assess stability, with poor stability often indicating higher risks of nonunion or malunion.

### 3D virtual surgical planning and patient-specific tools

After 3D visualization and biomechanical simulation deepen understanding of scaphoid fractures, the preoperative workflow reaches its most direct stage: translating therapeutic concepts into an executable surgical plan. Virtual surgical planning (VSP) allows surgeons to rehearse procedures on a computer, whereas 3D printing converts the plan into tangible intraoperative tools, with the aim of improving technical accuracy, promoting workflow standardization, and supporting individualized treatment in selected cases.

#### Virtual surgical planning

The core of VSP is patient-specific 3D modeling for preoperative surgical rehearsal, typically including simulated fragment reduction, screw-trajectory planning, implant sizing, and anticipation of intraoperative challenges. Murase *et al.* (36) showed that 3D computer simulation facilitates understanding of complex deformities in scaphoid nonunion and may improve corrective outcomes, supporting early clinical adoption of VSP. In scaphoid fracture management, VSP most commonly optimizes screw placement. Ideal insertion aligns with the central scaphoid long axis, is as perpendicular as possible to the fracture line, maximizes purchase through greater length, and avoids joint penetration and injury to surrounding critical structures, goals that are difficult to achieve simultaneously with freehand techniques or 2D fluoroscopic guidance. Accordingly, key parameters such as the long axis and safe screw corridor have been derived from measurements of large 3D-reconstructed scaphoid datasets (37, 38), providing anatomical constraints for VSP. VSP also enables iterative testing of entry points and insertion angles with real-time visualization of screw–bone relationships; comparative computational analyses have evaluated screw positions and explored approaches that better approximate fracture-perpendicular fixation (39, 40). More recently, multi-objective constrained path-planning algorithms have optimized trajectories for maximizing screw length, enhancing compressive effect across the fracture, and maintaining distance from the articular surface (41), indicating a shift from visualization toward intelligent planning that may proactively propose operative strategies.

#### Preoperative applications of 3D printing

3D printing (additive manufacturing) fabricates physical objects from digital 3D models layer by layer and has two main preoperative applications in scaphoid fracture management. First, 1:1 anatomical models can extend VSP: tangible models improve understanding of fracture morphology and spatial relationships (particularly for junior surgeons) and allow simulation of reduction maneuvers and instrument trajectories. Second, patient-specific external immobilization splints can be produced for nonoperative care or while awaiting fixation. Tobler-Ammann *et al.* (42) described a design and fabrication workflow for 3D-printed wrist splints for scaphoid and distal radius fractures and, in a randomized feasibility trial versus conventional fiberglass casts, reported superior acceptability and safety, supporting clinical translation. However, the use of patient-specific jigs or guides in scaphoid fracture fixation is technically demanding in practice. The scaphoid provides only a small and irregular contact surface, the percutaneous working corridor is narrow, and surrounding soft tissues may limit accurate guide seating. Even minor mismatch between the preoperative model and the intraoperative position can compromise trajectory accuracy. Ongoing work is therefore focused on optimizing guide footprint, fixation stability, registration reliability, and intraoperative verification.

## Intraoperative management

### Computer-assisted scaphoid surgery

Percutaneous screw fixation is a minimally invasive option for selected scaphoid fractures, particularly nondisplaced or minimally displaced scaphoid waist fractures. However, the complex 3D anatomy and confined operative space of the scaphoid make accurate implant placement challenging. Conventional fluoroscopy-guided fixation relies on repeated guidewire insertions, leading to increased radiation exposure, unnecessary bone loss, and potential compromise of fracture healing (43).

Computer-assisted surgery (CAS) has emerged to address these limitations, serving as a foundational digital technology that bridges preoperative imaging with intraoperative execution. It has evolved through 2D fluoroscopic navigation, 3D imaging guidance, and electromagnetic systems (43, 44, 45). Early foundational work established core principles such as image-to-patient registration (46). Initial cadaver studies of 2D fluoroscopic navigation demonstrated a significant reduction in radiation exposure and the number of guidewire attempts compared with conventional methods, although without consistently improving final screw positional accuracy (43, 47, 48).

Subsequent technological optimization focused on overcoming the inherent limitations of 2D imaging. The integration of 3D navigation with modalities such as cone-beam CT (CBCT) or intraoperative CT (O-arm) provided superior volumetric guidance (44, 49, 50, 51, 52). These systems demonstrated more consistent central screw placement, fewer drilling attempts, and maintained radiation reduction benefits. Furthermore, electromagnetic navigation systems (ENS) were introduced, virtually eliminating intraoperative fluoroscopy and showing high precision in screw alignment, albeit requiring an additional incision for sensor placement (45).

Despite these advantages, CAS for the scaphoid faces persistent hurdles. Registration inaccuracy, caused by even minor movement of the scaphoid relative to tracking references, remains a critical source of error (53). The technology still fundamentally depends on the surgeon’s manual dexterity and hand–eye coordination to follow the planned trajectory on a screen, with hand tremors potentially causing significant deviations in the small bone. Other barriers to widespread adoption include high equipment costs, added setup time, and a scarcity of large-scale clinical trials, with most evidence derived from cadaveric or small *in vitro* studies. CAS offers measurable benefits over traditional techniques, primarily through a marked reduction in radiation exposure and more efficient screw placement. However, to realize its full potential, challenges related to registration reliability, cost, and dependence on manual execution must be addressed (53).

### Robot-assisted scaphoid surgery

Building upon the foundation of computer-assisted navigation, robot-assisted surgery represents a significant evolution in digital orthopedics for scaphoid fracture management. It directly addresses the core limitations of manual execution by offering enhanced mechanical stability, mitigating hand tremor, and providing precise execution of preplanned trajectories through a rigid robotic arm. Current clinical evidence is predominantly derived from studies using the TiRobot orthopedic surgical robot system (TINAVI Medical Technologies Co., Ltd., China) and spans selected scaphoid pathologies. Typical robotic procedures are demonstrated in Fig. 3.

**Figure 3:**
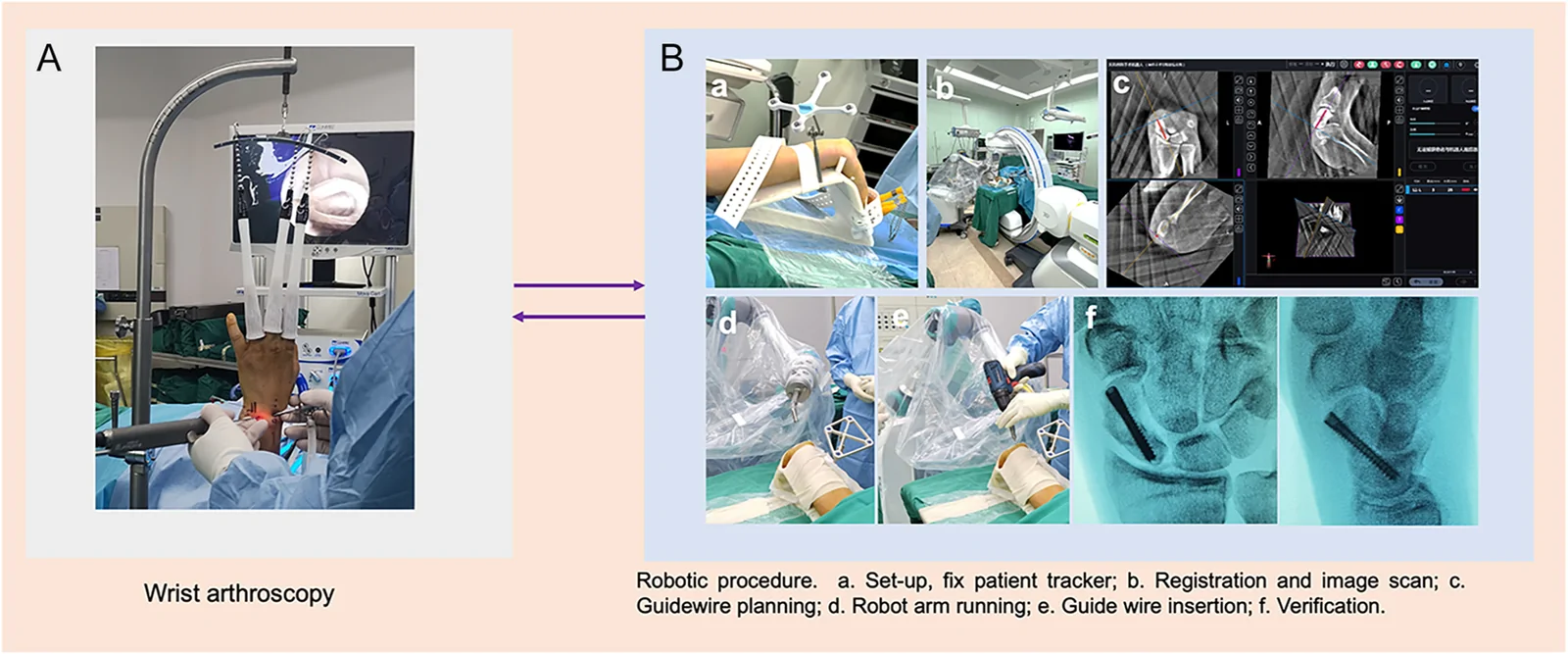
Robot-assisted scaphoid surgery procedure. Except for acute nondisplaced scaphoid fractures, wrist arthroscopy is typically performed both prior to robotic execution for debridement, fracture reduction, and bone grafting and subsequently to confirm the quality of the reduction and graft placement.

#### Acute scaphoid fractures

For acute scaphoid fractures, robotic assistance has shown potential benefits in technical precision and efficiency (54, 55, 56, 57). Studies involving acute, nondisplaced, or minimally displaced waist fractures report high rates of successful guidewire placement on the first attempt, significantly reduced intraoperative fluoroscopy, and superior screw alignment accuracy compared with freehand techniques (55, 56). The pioneering clinical application was reported by Liu *et al.* in 2019, involving 10 patients with acute waist fractures and establishing the initial feasibility and safety profile (54). Subsequent studies, including a randomized controlled trial by Guo *et al.* (55), corroborated these findings, demonstrating high rates of successful guidewire placement on the first attempt, significantly reduced intraoperative fluoroscopy, and superior screw alignment accuracy compared with freehand techniques. While operative setup time may be longer, the actual guidewire insertion time is often shorter (55). Clinical outcomes, including union rates (typically around 8–9 weeks) and functional scores (e.g. Mayo Wrist Score, Patient-Rated Wrist Evaluation), are excellent and comparable to conventional freehand methods. Notably, a 2025 double-center study by Yi *et al.* on 62 patients provided the first medium-term follow-up data (mean 36 months) and a learning curve analysis, confirming the technique’s reproducibility and excellent sustained outcomes after an initial learning phase of 10–20 cases (57).

#### Scaphoid nonunion

In the treatment of scaphoid nonunion, robotic technology has been used alone or integrated with advanced techniques such as arthroscopic bone grafting (58, 59, 60, 61). This combination allows for minimally invasive grafting and highly accurate screw fixation, even in challenging cases such as proximal pole fractures (59). Robotic guidance ensures optimal screw placement, reducing the loss of healthy bone mass and contributing to high union rates. Studies, including one by Guo *et al.* in 2020 on double-screw fixation without grafting for delayed unions, showed that robotic guidance ensured optimal screw placement through often sclerotic bone, contributing to high union rates (58). Recent innovations, such as combining robotic fixation with pulsed CO_2_ gas arthroscopy for grafting, have enhanced graft compaction compared with dry arthroscopy, highlighting the role of robotics in evolving, less invasive surgical strategies for nonunion (60, 61).

#### Other scaphoid-related injuries

Robotic applications are also extending to other complex scaphoid-related injuries. Preliminary case series reports or cadaveric studies describe successful integration with arthroscopic reduction for trans-scaphoid perilunate fracture–dislocations and with limited carpal fusions for advanced scaphoid nonunion collapse (SNAC wrist) (62, 63, 64). These reports underscore its potential utility in complex trauma and salvage procedures for scaphoid surgery where precision is paramount.

Cadaveric studies provide foundational evidence for the robotic system’s technical performance, consistently demonstrating a drastic reduction in the number of guidewire attempts (often to a single attempt), lower radiation exposure for the surgeon, and high positional accuracy of implants (64, 65, 66). These studies confirm the system’s ability to overcome the high technical dependency associated with conventional percutaneous fixation (Table 3).

**Table 3:** Summary of clinical studies on robot-assisted scaphoid surgery.

Study	Patients, *n*	Diagnosis	Robot-assisted procedure	Key outcomes	LOE
Liu *et al.* (54)	10	Acute undisplaced waist fractures	TiRobot (3D image)-assisted single-screw fixation	Mean union 8 weeks, MWS 96, PRWE 2; grip strength 91% of contralateral	IV
Guo *et al.* (58)	12	Minimally displaced waist nonunion/delayed union	TiRobot (2D image)-assisted double screws; no graft	Mean union 8 weeks, VAS 2; grip strength improved 30%; flexion-extension improved 20%	IV
Guo *et al.* (55)	36 (18/group)	Acute minimally displaced Herbert B2 fractures	TiRobot (2D image) vs freehand double screws	Both groups mean union at 8 weeks; similar ROM, grip strength, VAS, and MWS	II
Xiao *et al.* (56)	18 (9/group)	Acute nondisplaced fractures	TiRobot (2D image) vs freehand single-screw fixation	Lower screw angle deviation with robot; similar union time and MWS	III
Yi *et al.* (63)	2	Trans-scaphoid perilunate fracture dislocation	Arthroscopy-assisted reduction and TiRobot (3D image)-assisted fixation	Union at 8 and 12 weeks; satisfactory pain and functional outcomes	IV
Yi *et al.* (59)	6	Scaphoid nonunion (4 waist, 2 proximal pole)	Arthroscopic bone graft + TiRobot (3D image)-assisted fixation	Mean union 13 weeks; VAS, QuickDASH, MWS, PRWE, grip and ROM improved	IV
Gao *et al.* (62)	1	Stage II SNAC	Arthroscopy-assisted four-corner fusion + TiRobot (3D image)-assisted fixation	Union 12 weeks; VAS 0; PRWE 6; QuickDASH 2.3; MWS 85	IV
Zhou *et al.* (61)	18	Scaphoid nonunion(12 waist, 2 distal pole, 4 proximal pole)	CO_2_ gas arthroscopy + iliac bone graft + TiRobot (3D image)-assisted fixation	Union 100% by 6 months; 83.3% excellent MWS; mean grip strength 36 kg, pinch strength 9 kg	IV
Yi *et al.* (57)	62	Acute non-/minimally displaced waist fractures	TiRobot (3D image)-assisted single-screw fixation	First-attempt acceptable guidewire 60/62; mean union 9 weeks; Mean PRWE 1.6, VAS: 0.4, MWS 96.3	IV

MWS, Mayo Wrist Score; PRWE, Patient-Rated Wrist Evaluation; ROM, range of motion; SNAC, scaphoid nonunion advanced collapse; VAS, pain visual analog scale; LOE, level of evidence.

Robot-assisted surgery for scaphoid fractures offers demonstrable advantages in procedural consistency, reduction of radiation exposure, and implant accuracy. Current evidence, though promising and growing, remains largely at Level IV. Challenges include longer setup times, high capital costs, and the need for specialized training. Future higher-quality comparative studies are needed to definitively establish its clinical superiority and cost-effectiveness relative to advanced navigation alone (57). Importantly, although the TiRobot system has been gradually introduced into multiple clinical centers across China with expanding subspecialty applications, the current evidence specifically addressing its role in the percutaneous fixation of scaphoid fractures remains predominantly derived from a single pioneering institution. Therefore, while the preliminary results are promising, the generalizability of these findings may be limited, and further multicenter, large-sample studies are urgently needed to validate the reliability and reproducibility of this robotic technique in diverse clinical settings.

### 3D printing in scaphoid fracture management

3D printing, or additive manufacturing, represents a versatile pillar of digital orthopedics, translating patient-specific anatomical data into tangible surgical tools that bridge the gap between virtual planning and operative execution (67). Based on preoperative CT imaging, this technology creates physical models and guides that bridge the gap between virtual planning and operative execution (68). Its applications in scaphoid surgery are multifaceted, primarily categorized into four parts: patient-specific percutaneous guides, reduction templates, 1:1 anatomical models, and custom-made prostheses, each addressing distinct surgical challenges (68).

#### Patient-specific guides

Patient-specific percutaneous guides are the most extensively studied application. These sterilizable, single-use devices are designed to fit uniquely onto the scaphoid tubercle or distal pole, providing a preplanned trajectory for guidewire insertion. Cadaveric and clinical studies consistently demonstrate their efficacy in enhancing procedural efficiency and reducing radiation exposure (69, 70, 71, 72, 73, 74, 75, 76, 77). For instance, Salabi *et al.* in 2019 conducted a paired cadaver study showing that 3D-printed guides significantly improved screw angular accuracy (1.6° and 3.3° vs 13.8° and 9.2° in freehand) and reduced fluoroscopic images by over 70% (70). Marcano-Fernández *et al.* reported a clinical series in which guide use shortened surgery time by 43% and fluoroscopy time by 52% compared with conventional freehand technique (72). The pinnacle of efficiency was shown by DeWolf *et al.*, whose cadaveric model achieved zero fluoroscopy use and a single guidewire attempt with a 3D-printed guide (74). The primary limitation is the static nature of these guides, which relies on perfect congruence between the preoperative CT anatomy and the intraoperative soft-tissue envelope.

#### Reduction templates

For complex displaced scaphoid fractures, nonunion, or malunions, 3D-printed reduction templates offer a solution for precise anatomical restoration (78, 79). These tools physically contour bone grafts and guide fragment alignment. A comparative study by Schweizer *et al.* found that using reduction templates resulted in superior correction of the humpback deformity (mean residual displacement 7° vs 26°) and higher union rates compared with freehand surgery (78). A 2025 comparative study by Wirth *et al.* demonstrated that 3D-printed reduction guides achieved high union rates (92% primary, 87% revision) and restored grip strength (85% of contralateral) in scaphoid nonunion reconstructions. However, as a retrospective series with short-term follow-up and heterogeneous graft selection, the evidence remains preliminary, highlighting the need for controlled long-term studies (79). These templates transform complex 3D spatial problems into simpler, tactile tasks but add to preoperative planning time and cost.

#### Anatomical scaphoid models

1:1 anatomical scaphoid models serve as valuable preoperative planning and communication tools (36, 80, 81, 82). Their utility is primarily in the planning phase, enhancing surgeon preparedness without providing direct intraoperative guidance. Early work by Murase *et al.* (2005) utilized stereolithography models to plan and execute corrections for scaphoid malunions (36). Schmidt *et al.* (2020) used a 3D-printed scaphoid model with a handlebar to precisely guide the harvest and contouring of a medial femoral condyle osteochondral flap, enhancing intraoperative accuracy for complex proximal pole reconstructions (80). Scaphoid models allow surgeons to physically handle a replica of the fracture, preselect and contour implants, and simulate the procedure. Studies highlight their role in optimizing plate bending for nonunion fixation and improving spatial understanding for proximal pole fractures. Their utility is primarily in the planning phase, enhancing surgeon preparedness but not providing direct intraoperative guidance.

#### Custom prostheses

In cases of SNAC or avascular necrosis where reconstruction is not feasible, 3D-printed custom prostheses present a motion-preserving salvage option (83, 84, 85, 86, 87, 88, 89). Made from biocompatible materials such as titanium alloy or polyetheretherketone (PEEK), these implants aim to restore near-normal carpal kinematics. Rossello (2020, 2021) reported promising early outcomes with both total and partial titanium scaphoid replacements, showing maintained range of motion and grip strength at 1–2 years postoperatively (85, 87). Larger series, such as that by Cioffi *et al.* (2023), indicated significant pain relief and functional improvement, albeit with reports of implant-related complications such as stem dislocation, underscoring the need for long-term follow-up (88). Kemler (2025) reported a bilateral case in which 3D-printed titanium scaphoid prostheses effectively treated scapholunate advanced collapse wrist, preserving grip strength and reducing pain at short-term follow-up, although long-term durability requires further investigation (Table 4) (89). Custom-made 3D-printed carpal bone prostheses should be regarded as early, salvage-stage applications rather than established routine treatment. Long-term durability, implant wear, fixation, migration, maintenance of carpal kinematics, and particularly ligament stabilization or reconstruction remain unresolved. Further work is needed to determine how stable ligamentous support can be restored around these implants and whether encouraging short-term results can be reproduced over longer follow-up.

**Table 4:** Summary of clinical studies on 3D printing-assisted scaphoid surgery.

Category/ Study	Sample / indication	3D-printed tool / application	Key findings
Patient-specific percutaneous guides			
Yin *et al.* (69)	Technique report	CT-based patient-specific guide for volar K-wire insertion	‘1-shot’ guidewire placement; reduced fluoroscopy
Wan *et al.* (71)	6 cadaveric limbs + 4 scaphoid fracture patients	Printed guide for volar percutaneous screw fixation	Clinical guidewire placement in 1 attempt; 100% union
Yin *et al.* (73)	16 patients (8 guide group, 8 freehand); minimally displaced/non-displaced nonunion	Guide plate(3 K-wires) + arthroscopic bone graft	Shorter bone-operation time; 100% union; similar functional outcomes vs freehand
Marcano-Fernández *et al.* (72)	20 patients (10 guide group,10 freehand); minimally displaced Herbert B1/B2 fractures	Guide-assisted volar percutaneous screw fixation	Similar screw accuracy; 43% shorter surgery and 52% less fluoroscopy
Lin *et al.* (75)	2 patients; Herbert B2 fractures	Guide-assisted dorsal percutaneous screw fixation	1-attempt insertion; surgery time less than 20 min
Rong *et al.* (76)	10 patients; minimally displaced waist fractures	Guide-assisted volar percutaneous screw fixation	1-attempt insertion; 100% union; improved grip strength and MMWS
Reduction templates			
Schweizer *et al.* (78)	22 patients; scaphoid nonunion	Patient-specific reduction template for open reconstruction	Union 8/9 vs 11/13 freehand; less residual displacement and shorter surgery time
Wirth *et al.* (79)	54 patients; 39 primary, 15 revision nonunion	Reduction-template-assisted open reconstruction	Union 92% primary and 87% revision; similar functional outcomes
1:1 anatomical models			
Murase *et al.* (36)	7 patients; scaphoid nonunion	1:1 model for planning of open reconstruction	Improved carpal alignment; 100% union; wrist score 89 (Cooney scale)
Jew *et al.* (81)	4 patients; complex fractures	1:1 model for fracture visualization and implant planning	Improved visualization/planning; reduced operation time; 100% union
Schmidt *et al.* (80)	2 patients; AVN proximal pole pseudarthrosis	Model for medial femoral condyle osteochondral flap harvest	Facilitated osteotomy and graft-recipient matching; accurate flap fit
Oki *et al.* (82)	1 patient; scaphoid nonunion	Model for plate fixation and iliac graft	Restored scaphoid morphology; improved capitolunate angle; DASH 7/100
Custom prostheses			
Rossello (85)	1 patient; nonunion with necrosis	TiNbN-coated titanium total scaphoid replacement + SLIL reconstruction	Improved grip strength; no SNAC progression
Rossello (87)	1 patient; nonunion with proximal pole necrosis	TiNbN-coated partial scaphoid replacement + SLIL reconstruction	Improved grip strength; return to competition
Cioffi *et al.* (88)	19 patients; nonunion with proximal necrosis/irreparable bone damage	Partial/total TiNbN-coated scaphoid replacement + SLIL reconstruction	VAS 0 at rest; DASH 9.2 partial vs 11.8 total; 2/19 dislocations
Kemler (89)	1 patient, bilateral wrists SLAC	TiAl6V4/TiNbN custom prosthesis with FCR tendon fixation	Marked pain reduction; grip strength maintained; no dislocation/periprosthetic arthritis

MMWS, modified Mayo Wrist Score; VAS, visual analog scale; DASH, Disabilities of the Arm, Shoulder and Hand; AVN, avascular necrosis; FCR, flexor carpi radialis; SLAC, scapholunate advanced collapse; SLIL, scapholunate interosseous ligament; SNAC, scaphoid nonunion advanced collapse.

## Limitations and challenges of digital orthopedic techniques

The clinical integration of digital orthopedic techniques for scaphoid fractures remains constrained by limitations in evidence quality, technical robustness, and real-world implementation. First, although AI-based diagnostic models have shown promising accuracy, the available evidence is still heterogeneous, with differences in datasets, imaging protocols, and validation strategies. Issues related to external generalizability, interpretability, regulatory oversight, and medico-legal responsibility remain incompletely resolved (11, 15, 16, 17, 54, 65, 69, 78). Second, advanced preoperative tools such as 3D reconstruction, fracture mapping, and FEA depend heavily on image quality, segmentation accuracy, and model assumptions, and their biomechanical outputs have not yet been consistently validated against intraoperative findings or long-term clinical outcomes (27, 28, 29, 30, 31, 32, 33, 34, 35) Third, VSP and 3D printing improve personalization, but they remain largely static and preoperative, with limited adaptability to unexpected intraoperative changes; in addition, their effectiveness is influenced by software–hardware interoperability, surgeon experience, and added planning time (36, 37, 38, 39, 40, 41, 42, 72). Finally, for intraoperative navigation, robotic techniques, and 3D printing, registration errors, workflow complexity, prolonged setup, and substantial equipment costs continue to hinder widespread adoption (54, 57, 65, 69, 78, 86, 88). Overall, broader clinical translation will require standardized workflows, high-quality comparative studies, and cost-conscious integration across the entire diagnostic and therapeutic pathway.

## Prospects and conclusion

Digital orthopedics is reshaping the management of scaphoid fractures from conventional image-based assessment toward a more precise, data-driven, and individualized paradigm, as shown in Fig. 4. In the preoperative setting, AI-assisted image interpretation, 3D reconstruction, fracture mapping, FEA, and VSP have expanded the ability to characterize fracture morphology, occult injury, biomechanical stability, and fixation strategy (7, 12, 13, 20, 25, 27, 28, 29, 30, 31, 32, 33, 34, 35, 36, 37, 38, 39, 40, 41). Intraoperatively, navigation (43), robotics (54), patient-specific guides (68), and other emerging digital tools have further improved the accuracy and reproducibility of reduction and fixation. An overview of digital orthopedic techniques for scaphoid fractures is shown in Fig. 4. Looking forward, the greatest potential will likely lie not in any single technology but in multimodal integration across the full care pathway, with closer convergence of intelligent diagnosis, patient-specific planning, real-time guidance, and precision execution. In particular, augmented reality/mixed reality (AR/MR)-based navigation and ultrasound-guided techniques may further enhance intraoperative visualization while reducing radiation exposure, especially when combined with AI-assisted segmentation and preoperative imaging fusion (90, 91, 92, 93, 94, 95).

**Figure 4:**
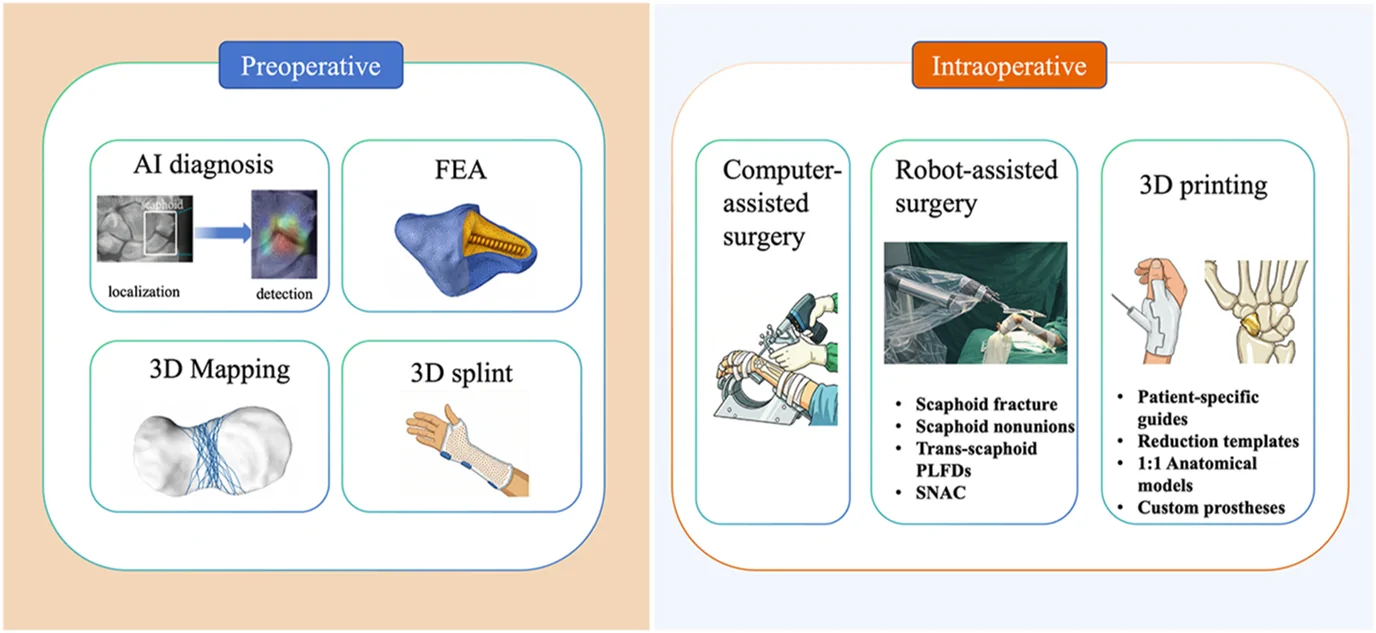
Overview of digital orthopedic techniques for scaphoid fractures. The left panel shows preoperative applications, including AI-based diagnosis, finite element analysis (FEA), 3D mapping, and 3D splint design. The right panel shows intraoperative applications, including computer-assisted surgery, robot-assisted surgery, and 3D printing-assisted procedures. Some illustrative components were generated with the assistance of artificial intelligence and were subsequently reviewed and refined by the authors for anatomical and technical accuracy.

Nevertheless, widespread adoption still requires stronger evidence, standardized workflows, interoperable platforms, cost-effectiveness data, and structured training systems (11, 15, 16, 17, 22). Current limitations in validation, workflow complexity, and economic burden should not be underestimated. Even so, the overall trajectory is clear: as technical barriers are progressively overcome and clinical evidence matures, digital strategies are likely to become an increasingly important component of modern scaphoid fracture care.

## ICMJE Statement of Interest

The authors declare that there is no conflict of interest that could be perceived as prejudicing the impartiality of the work reported.

## Funding Statement

This study was supported by the National Natural Science Foundation of China (82272581), the Beijing Natural Science Foundation–Haidian Original Innovation Joint Fund Project (L252124), the Beijing Municipal Health Commission (BJRITO-RDP-2024), the Beijing Hospitals Authority’s Ascent Plan (DFL20240402) and the FY 2026–2027 The Belt and Road Health International Cooperation Project and World Health Organization Collaborating Centre Project of Beijing Municipal Health Commission.

## Author contribution statement

All authors had full access to the data in the study and take responsibility for the integrity of the data and the accuracy of the data analysis. BL conceptualized the study. SW, ZY, and WC contributed to methodology, validation, investigation, formal analysis, and resource acquisition and prepared the original draft. SW, ZY, WC, and BL contributed to review and editing. SW, ZY, and WC performed visualization. BL supervised the study.

## Data availability

The data that support the findings of this study are available from the corresponding author upon reasonable request.
